# An Integrated Miniature Bioprocessing for Personalized Human Induced Pluripotent Stem Cell Expansion and Differentiation into Neural Stem Cells

**DOI:** 10.1038/srep40191

**Published:** 2017-01-06

**Authors:** Haishuang Lin, Qiang Li, Yuguo Lei

**Affiliations:** 1 Department of Chemical and Biomolecular Engineering, University of Nebraska, Lincoln, Nebraska, USA; 2 Mary and Dick Holland Regenerative Medicine Program, University of Nebraska Medical Center, Omaha, Nebraska, USA; 3 Fred & Pamela Buffett Cancer Center, University of Nebraska Medical Center, Omaha, Nebraska, USA

## Abstract

Human induced pluripotent stem cells (iPSCs) are ideal cell sources for personalized cell therapies since they can be expanded to generate large numbers of cells and differentiated into presumably all the cell types of the human body *in vitro*. In addition, patient specific iPSC-derived cells induce minimal or no immune response *in vivo*. However, with current cell culture technologies and bioprocessing, the cost for biomanufacturing clinical-grade patient specific iPSCs and their derivatives are very high and not affordable for majority of patients. In this paper, we explored the use of closed and miniature cell culture device for biomanufacturing patient specific neural stem cells (NSCs) from iPSCs. We demonstrated that, with the assist of a thermoreversible hydrogel scaffold, the bioprocessing including iPSC expansion, iPSC differentiation into NSCs, the subsequent depletion of undifferentiated iPSCs from the NSCs, and concentrating and transporting the purified NSCs to the surgery room, could be integrated and completed within two closed 15 ml conical tubes.

Cell therapies offer hope to treat many human diseases and injuries that cannot be treated or their progression cannot be altered by current treatments^1^. Autologous cells are ideal for cell therapies since they induce minimal or no immune rejection^2^,^3^. However, it is very challenging to isolate sufficient numbers of autologous cells from the patient for an effective treatment^4^. Furthermore, majority of human cell types exhibit limited growth and significant phenotype shifting when cultured, preventing generating the required numbers of autologous cells through *in vitro* expansion^1^,^2^,^3^,^4^,^5^. Human induced pluripotent stem cells (iPSCs) provide a solution for this challenge. With reprogramming factors, adult cells from the patient, such as fibroblasts, can be reprogrammed into iPSCs within about one month^6^. iPSCs can be cultured for long term and expanded into large numbers under complete defined conditions^7^. They can be differentiated into presumably all the cell types of the human body^2^,^8^,^9^. During the past decade, protocols for efficiently differentiating iPSCs into various human cell types, such as cortical neurons^10^,^11^, gamma-aminobutyric acid (GABA)-ergic interneurons^12^,^13^,^14^, midbrain dopaminergic (DA) neurons^15^,^16^, endothelial cells^17^,^18^,^19^, mesenchymal stem cells^20^,^21^, cardiomyocytes^22^,^23^,^24^, hepatocytes^25^,^26^,^27^, beta cells^28^,^29^ and other cells^2^,^8^,^9^ have been developed. Many of these cells are being investigated for treating degenerative diseases and injuries^30^, such as Parkinson’s disease (PD)^15^,^16^,^31^, Alzheimer’s disease (AD)^32^, stroke^33^, spinal cord injury (SCI)^34^,^35^,^36^,^37^, blindness^8^,^38^,^39^, myocardial infarction (MI)^22^,^40^, diabetes etc. The iPSC-derived retinal pigment epithelium has been tried in human^8^. In short, iPSCs are ideal cell sources for personalized cell therapies.

However, the advancement of iPSC-based personalized cell therapies is currently hindered by the high cost to biomanufacture the cells^1^,^2^,^3^,^4^,^5^. With the current bioprocessing^41^, patient cells are collected and cultured for a few days^41^; then, reprogramming factors are delivered to these cells to reprogram them into iPSCs (which takes approximately one month). Next, high quality iPSC clones are selected, expanded and characterized for their pluripotency and genome integrity with a variety of assays (which takes approximately one to two months); then, iPSCs are expanded and differentiated into the desired cells. Finally, the produced cells are purified, characterized for their identities, purity, and potency and formulated for transplantation. The whole bioprocessing takes a few months and is mainly done using 2D, open culture systems (e.g., 2D cell culture flasks) through manual operations–a processing which leads to low reproducibility, high risk of contamination, and requirement for highly skilled technicians^42^. The whole bioprocessing is also required to comply with the current Good Manufacturing Practice (cGMP)^42^. In addition, 2D culture systems have low yield. For instance, only ~2 × 10^5^ cells can be produced per cm^2^ surface area, meaning that it will require ~85 six-well plates to produce the cells (~1 × 10^9^ cells) sufficient for one patient^43^,^44^. Maintaining these plates requires large incubator and cGMP-compliant facility space, labor, and reagent. If large numbers of patients need iPSC-based personalized cell therapies, the cell production can only be done in large cell biomanufacturing centers (i.e. the centralized cellular biomanufacturing)^42^. Patient cells are sent to the center, and the produced cells are sent back to the point-of-care for transplantation. This centralized biomanufacturing has additional disadvantages^1^,^42^,^45^, including: (i) patient cells may be cross-contaminated and (ii) there are high costs and risks associated with the transportation, logistics, tracking, and recording. In summary, the cost for biomanufacturing personalized iPSCs and their derivatives with current technologies is not affordable for the majority of patients^1^,^2^,^3^,^4^,^5^.

One method to significantly reduce the biomanufacturing cost is to make cells in individualized, closed, computer controlled miniature cell culture device at the point-of-care (i.e. the cGMP-in-a-box production)^42^. Using closed culture devices avoids contamination risk and eliminates the requirement for cGMP processing. Automation of all key operations avoids output variations and reduces need for highly skilled operators. Biomanufacturing at the point-of-care reduces the cost and risk related to the logistics and transportation. Miniaturizing the culture system makes it possible to simultaneously biomanufacture cells for large numbers of patients at the point-of-care (i.e. high throughput biomanufacturing).

In this paper, we describe our effort to develop such a miniature bioprocessing for making NSCs from human iPSCs. The bioprocessing takes advantage of the discovery that human iPSCs could be expanded in 3 dimension (3D) thermoreversible Poly(N-isopropylacrylamide)-Poly(ethylene glycol) (PNIPAAm-PEG) hydrogels at high growth rate and yield^43^,^46^. In this paper, we first developed a protocol that could efficiently differentiate human iPSCs into NSCs in the PNIPAAm-PEG hydrogel. We then, with the assist of this hydrogel scaffold, integrated the bioprocessing including the iPSC expansion, iPSC differentiation into NSCs, the subsequent depletion of undifferentiated iPSCs from the product, and concentrating and transporting the produced cells to the surgery room into two closed, 15 ml conical tubes.

## Methods

### Culturing human pluripotent stem cells (hPSCs) in 2D

iPSCs (iPSCs reprogrammed from human mesenchymal stem cells) were obtained from George Q. Daley laboratory (Children’s Hospital Boston, Boston)^47^. H9 hESCs were purchased from WiCell Research Institute. hPSCs (iPSCs and H9s) were maintained in 6-welll plate coated with Matrigel (BD Biosciences) in Essential 8^TM^ medium (E8, Invitrogen)^7^. Cells were passaged every 4 days with 0.5 mM EDTA (Invitrogen). Medium was changed daily. Cells were routinely checked for the expression of pluripotency markers, OCT 4 and NANOG, their capability to form teratomas in immunodeficient mice, their karyotypes and bacterial contaminations.

### Culturing hPSCs in 3D PNIPAAm-PEG hydrogels

To transfer the culture from 2D to 3D PNIPAAm-PEG hydrogels, hPSCs maintained in Matrigel-coated 6-welll plate were treated with Accutase (Life Technologies) at 37 °C for 5 minutes and dissociated into single cells^43^,^46^. Dissociated cells were mixed with 10% PNIPAAm-PEG (Cosmo Bio, USA) solution dissolved in E8 medium on ice and cast on tissue culture plate, then incubated at 37 °C for 10 minutes to form hydrogels before adding warm E8 medium containing 10 μM ROCK inhibitor (Y-27632, Selleckchem). Medium was changed daily. Cells were passaged every 5 days. To passage cells, medium was removed, and 2 ml ice-cold PBS was added to dissolve the hydrogel for 5 minutes. Cell spheroids were collected by spinning at 100 g for 3 minutes. Cells were incubated in Accutase (Invitrogen) at 37 °C for 10 minutes and dissociated into single cells.

### hPSC differentiation in 2D

Single hPSCs (iPSCs and H9s) were plated in Matrigel-coated 6 well plates (2 × 10^6^ cells/well) and cultured in E8 medium overnight to reach >90% confluency. E8 medium was removed and replaced with neural induction medium consisting of Essential 6^TM^ medium (E6, Invitrogen) supplied with 100 nM LDN193189 (Selleckchem) and 10 μM SB431542 (Selleckchem) for 7 days. Note that E6 medium is equal to E8 medium minus the bFGF and TGFβ proteins. Medium was changed daily^48^.

### hPSC differentiation in 3D PNIPAAm-PEG hydrogels

Single hPSCs were encapsulated in the PNIPAAm-PEG hydrogels (1 × 10^6^ cells/ml hydrogel) and cultured in E8 medium for 5 days. E8 medium was removed and replaced with neural induction medium for 7 days. Medium was changed daily. To differentiate NSCs into the cortical neurons, NSC spheroids were harvested on day 11 and plated to Matrigel-coated 6 well plates, and cultured in neural differentiation medium consisting of Neurobasal^®^ Media (Life Technologies), B27 (50X, Life Technologies), BDNF (20 ng/ml, PeproTech), GDNF (10 ng/ml, PeproTech), L-ascorbic acid (200 μM, Sigma), DAPT (2.5 μM, Tocris), Dibutyryl-cAMP (0.5 mM, Santa Cruz Biotechnology) for another 19 days. Half medium was changed every two days.

To make ventral midbrain NSCs, hPSC spheroids were cultured in neural induction medium containing 50% DMEM/F12 + 50% Neurobasal medium, 1% N2, 2% B27 minus vitamin A, SB431542 (10 μM), LDN193189 (100 nM), CHIR99021 (0.7 μM, Selleckchem), purmorphamine (2 μM, Selleckchem) and sonic hedgehog (shh) C25 II (200 ng/ml, Home-made) in the 3D PNIPAAm-PEG hydrogels for 11 days. NSC spheroids were harvested on day 11 and plated to Matrigel-coated 6 well plates, and cultured in neural differentiation medium consisting of Neurobasal^®^ Media (Life Technologies), B27 (50X, Life Technologies), BDNF (20 ng/ml, PeproTech), GDNF (10 ng/ml, PeproTech), L-ascorbic acid (200 μM, Sigma), DAPT (2.5 μM, Tocris), Dibutyryl-cAMP (0.5 mM, Santa Cruz Biotechnology) for another 19 days. Half medium was changed every two days.

### Culturing hPSC-derived NSCs in 2D

NSCs made from hPSCs were incubated with Accutase at 37 °C for 5 minutes and dissociated into single cells. Cells were plated on Matrigel-coated 6 well plate at high density (2.0 × 10^6^ cells/ml) and cultured in expansion medium consisting of KnockOut^TM^ DMEM/F-12 (Life Technologies), StemPro Neural Supplement (50X, ThermoFisher Scientific), 20 ng/ml bFGFs (Peprotech), 10 ng/ml EGFs (Peprotech), Glutamax I (100X, Gibco) and 2 μg/ml heparin (Sigma). Medium was changed daily and cells were passaged every 3 days.

### Culturing hPSC-derived NSCs in 3D PNIPAAm-PEG hydrogels

Dissociated single NSCs were encapsulated to 3D PNIPAAm-PEG hydrogels at 2 × 10^6^ cells/ml and cultured in expansion medium containing KnockOut^TM^ DMEM/F-12, StemPro Neural Supplement (50X, ThermoFisher Scientific), 20 ng/ml bFGFs (Peprotech), 10 ng/ml EGFs (Peprotech), Glutamax I (100X, Gibco) and 2 μg/ml heparin (Sigma). Medium was changed daily and cells were passaged every 7 days with Accutase.

### Integrated miniature bioprocessing

With a syringe and needle, 4 °C PNIPAAm-PEG solution containing single iPSCs were injected into room temperature E8 medium in a 15 ml conical tube. Fibrous hydrogels were formed instantly. A long needle was inserted through the septum cap into the tube that its open reached the tube bottom. A Variable-Speed Peristaltic Tubing Pump (Control Company, USA) was used to continuously perfuse the culture medium into the tube through this long needle. A second short needle was placed in the septum cap so that the medium can flow out. Medium was stocked in a sealed and oxygen-permeable plastic bag. Medium in the bag was changed daily. The cell culture tube, pump and medium bag were placed in a cell culture incubator at 37 °C. E8 medium and neural induction medium was used for day 1 to 5, and day 6 to 12, respectively. On day 12, the cell culture tube was placed on ice for 5 minutes to liquefy the hydrogel and release the spheroids. Cells were collected by spinning the tube at 100 g for 5 minutes. The cell pellet was treated with Accutase at 37 °C for 10 minutes and dissociated into single cells. Single cells were collected by spinning at 300 g for 5 minutes. Cells were re-suspended with 80 μl PBS buffer and 20 μl of anti-SSEA-4 microbeads (Miltenyi Biotec) were added and incubated at 4 °C for 15 minutes. The SSEA4+ iPSCs were pulled down with a magnet and NSCs in the supernatant were transferred into a new tube. Cells were pelleted by spinning at 300 g for 5 minutes and transported to the surgery room for transplantation.

### Transplanting iPSC-derived NSCs to rat brains

All animal protocols were approved by the Animal Care and Use Committee of the University of Nebraska, Lincoln. All experimental procedures involving animals were carried out in accordance with the guidelines of the Institutional Animal Care and Use Committee of the University of Nebraska, Lincoln. Sprague Dawley female rats were obtained from Charles River. Animals received intraperitoneal cyclosporine A (10 mg/kg, LC Laboratories) injection starting 1 day before transplantation. For transplantation, animals were anesthetized with 2–4% isoflurane. 2 × 10^5^ cells suspended in 4 μl DMEM medium were injected into the striatum (AP + 0.5 mm; ML ± 3.0 mm; DV-6 mm) at 0.5 μl/minute using a 10 μl Hamilton syringe (Hamilton Company, USA) with a stereotaxic frame (RWD Life Science Inc). On day 7, rats were anesthetized with ketamine/xylazine and perfused with PBS followed by 4% paraformaldehyde. After fixation, the brain was serially sectioned (40 μm in thickness) with a Leica cryosection machine, and free-floating sections were stained with antibodies.

### Immunostaining and imaging

Cells cultured on 2D surface were fixed with 4% paraformaldehyde (PFA) at room temperature for 15 minutes, permeabilized with 0.25% Triton X-100 for 10 minutes and blocked with 5% goat serum for 1 hour before incubating with primary antibodies at room temperature for 2 hours. After extensive wash, secondary antibodies in 2% BSA were added and incubated for another 1 hour.

**Figure 1:**
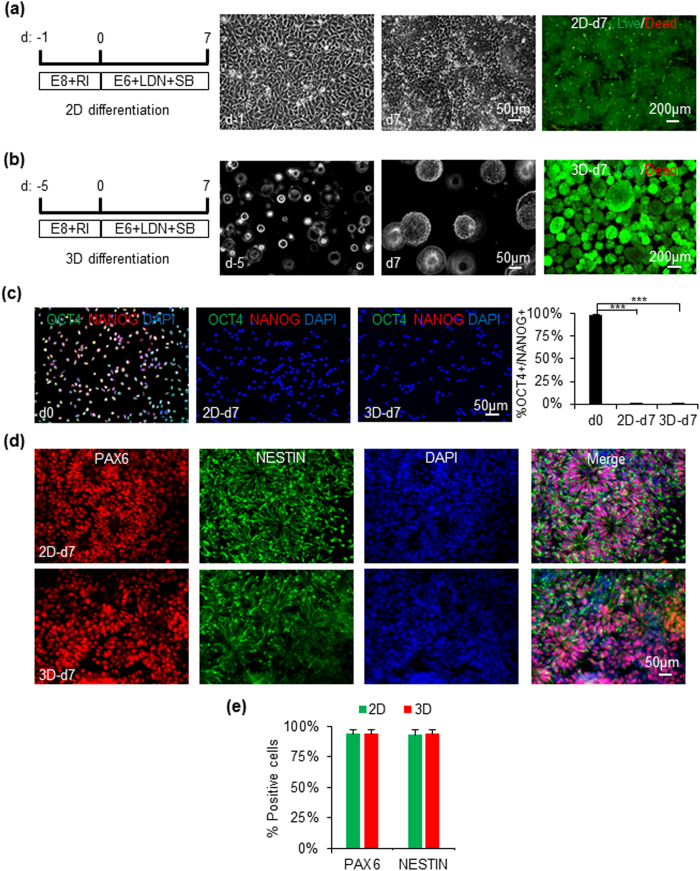
Differentiation of human iPSCs into NSCs in 2D cultures and 3D PNIPAAm-PEG hydrogels. For differentiation in 2D (**a**), single iPSCs were plated on Matrigel-coated plate overnight to reach 90% confluency. For differentiation in 3D (**b**), single iPSCs were cultured in the PNIPAAm-PEG hydrogel for 5 days to generate uniform spheroids. Cells were differentiated in neutral induction medium for 7 days. Phase images for cells on day 0 (d0) and 7 (d7), as well as live/dead cell staining for cells on day 7 are shown. (**c**) 98% of cells expressed the pluripotency markers, OCT4 and NANOG before the differentiation. Less than 2% of cells were OCT4+/NANOG+ after 7 days differentiation in 2D or 3D. Cells were dissociated into single cells and plated on Matrigel-coated plate for 6 hours before the fixation and staining. (**d**,**e**) On day 7, ~95% of cells were positive for the NSC markers, PAX6 and NESTIN. The 3D spheroids were plated on Matrigel-coated plate overnight to form monolayers before the fixation and staining. The triple asterisk (***) indicates statistical significance at a level of *p* < 0.001.

**Figure 2:**
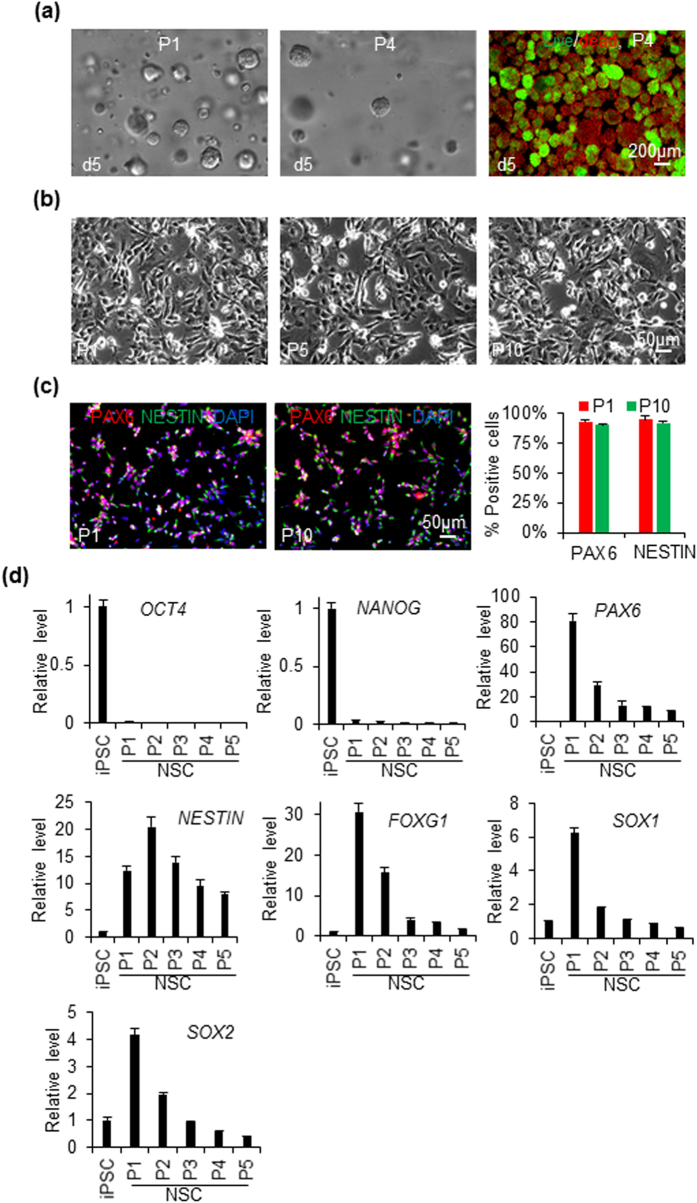
Culturing iPSC-derived NSCs in 3D PNIPAAm-PEG hydrogels and 2D cultures. NSCs derived from iPSCs in the 3D PNIPAAm-PEG hydrogel were dissociated into single cells and cultured in the 3D PNIPAAm-PEG hydrogel (**a**) or on Matrigel-coated 6 well plate (**b–f**). Cells were passaged every 5 days or 3 days for 3D and 2D cultures, respectively. (**a**) phase and live/dead staining images of day 5 spheroids at passage 1 (P1) and 4 (P4) in the 3D PNIPAAm-PEG hydrogels. (**b**) Phase images of NSCs on day 3 of passage 1, 5 and 10 (P1, P5, P10) in 2D cultures. (**c**) 90-95% of the NSCs at P1 and P10 in 2D cultures were positive for the NSC markers, PAX6 and NESTIN. (**d**) qPCR data on the expressions of pluripotency and NSC markers during P1 to P5 in 2D cultures.

**Figure 3:**
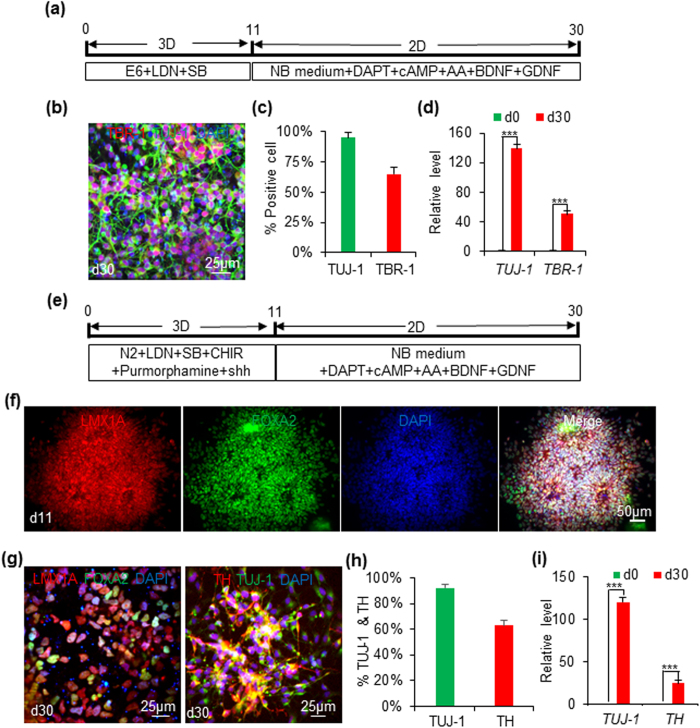
Differentiating iPSCs into cortical and ventral midbrain NSCs in 3D PNIPAAm-PEG hydrogels. (**a**) When iPSCs were differentiated in the E6 medium+ LDN193189+ SB431542 in the 3D PNIPAAm-PEG hydrogels for 11 days, and further differentiated in the neural differentiation medium in 2D for 19 days, cortical neurons could be made. 95% and 65% of the produced cells were positive for TUJ-1 and TBR-1, receptively (**b,c**). (**d**) qPCR showed the expressions of *TUJ-1* and *TBR-1* were increased 139 and 51-fold on day 30, respectively. (**e**) When iPSCs were cultured in the E6 medium + LDN193189 + SB431542 + CHIR99021 + shh + purmorphamine in the 3D PNIPAAm-PEG hydrogels for 11 days, they became FOXA2+ and LMX1A+ ventral midbrain progenitor cells (**f**). When they were further differentiated in the neural differentiation medium in 2D for 19 days, FOXA2+ and LMX1A+ ventral midbrain neurons could be made. 92% and 63% of the produced cells were positive for TUJ-1 and TBR-1, receptively (**g,h**). (i) qPCR showed the expressions of *TUJ-1* and *TH* were increased 120 and 25-fold on day 30, respectively. The triple asterisk (***) indicates statistical significance at a level of *p* < 0.001.

To assess the pluripotency of hPSCs expanded in 3D PNIPAAm-PEG hydrogels, hPSCs were dissociated into single cells with accutase, stained in suspension. Cells were placed in 96 well plate and imaged with Zeiss Axio Observer Fluorescent Microscopy. The percentage of OCT4+ or NANOG+ nuclei was quantified with Image J software. This method was also used to quantify the PAX6+ or NESTIN+, or TUJ-1+ or TBR-1+ cells in Figs 1, 2, 3 and 5. At least 1000 cells were analyzed. To stain the brain sections, samples were incubated with PBS+ 0.25% Triton X-100 + 5% goat serum+ primary antibodies at 4 °C for 48 hours. After extensive wash, secondary antibodies in 2% BSA was added and incubated at 4 °C for 4 hours.

LIVE/DEAD^®^ Cell Viability kit (Invitrogen) was used to stain the live and dead cells following the product manual. Antibodies were used: OCT4 (1:500, Santa Cruz Biotech), NANOG (1:200, Santa Cruz Biotech), PAX6 (1:300, Biolegend), NESTIN (1:1000, Biolegend), TUJ-1 (1:10,000, Sigma), TBR-1 (1:500, Abcam), TH (1:500, Pel-Freez), and GABA (1:1000, Sigma), HuNu (1:200, Millipore). Secondary antibody: Alexa Fluor 488-AffiniPure Donkey Anti-Mouse IgG, Cy3-AffiniPure Donkey Anti-Rabbit IgG.

### Quantitative PCR (qPCR)

Total RNA was extracted from the harvested cells using Trizol (Invitrogen, Carlsbad, CA, USA), according to the manufacturer’s instructions. Reverse transcription is done with the Maxima First Strand cDNA Synthesis Kit. Quantitative real-time PCR was carried out in an Eppendorf MasterCycler RealPlex4 (ThermoFisher Scientific) using the Power SYBR Green PCR Master Mix (ThermoFisher), according to the manufacturer’s instructions. The data were normalized to the endogenous GAPDH.

### Teratoma formation *in vivo*

All animal protocols were approved by the Animal Care and Use Committee of the University of Nebraska, Lincoln. All experimental procedures involving animals were carried out in accordance with the guidelines of the Institutional Animal Care and Use Committee of the University of Nebraska, Lincoln. 3 × 10^6^ hPSCs (small clumps) were suspended in 25 μl PBS + 25 μl Matrigel (BD Biosciences) and injected subcutaneously at the back of the neck of the NOD-SCID mice (Charles River). Teratoma was harvested when its size reached 2 cm. Teratoma was fixed with 4% PFA for 48 hours, dehydrated with 70%, 95% and 100% ethanol sequentially, and de-fated with xylene for 2 hours before embedded in paraffin. 10 μm thick section and stained was cut and stained with hematoxylin and eosin. The structures from all 3 germ layers were identified by trained specialist.

### Statistical analysis

Statistical analyses were done using the statistical package Instat (GraphPad Software, La Jolla, CA). For multiple comparisons, the means of triplicate samples were compared using the Tukey multiple comparisons analysis with the alpha level indicated in the figure legend.

## Results

The human iPSCs used for this study were made from human mesenchymal stem cells with retrovirus containing the OKSM (i.e. *OCT4, KLF4, SOX2*, and *c-MYC*) reprogramming factors^47^. They formed compact colonies when cultured on Matrigel-coated cell culture plates in the chemical defined Essential 8 (E8) medium. iPSCs expressed the pluripotency makers, OCT4 and NANOG. They could be differentiated into all the three germ layer cells, such the NESTIN+ ectodermal, α-SMA+ mesodermal and FOXA2+ endodermal cells in the embryoid body (EB) assay (Fig. S1). They formed teratomas after transplanting to the immunodeficient SCID mice. All the three germ layer tissues and cells were found in the teratomas (Fig. S1). To show that results found in this paper is general to human pluripotent stem cells (hPSCs), we also used the H9 hESCs for the research.

Both the iPSCs and H9s could be encapsulated and cultured in the thermoreversible PNIPAAm-PEG hydrogels for long term (Fig. S1)^43^,^46^. The aqueous 10% PNIPAAm-PEG polymer solution is liquid at low temperature (e.g. below 4 °C) and forms elastic hydrogel with storage modulus around 1000 Pa upon heating above room temperature. With E8 medium supplied with 10 μM ROCK inhibitor Y-27632, single iPSCs could survive and undergo clonal expansion into uniform spherical cell aggregates (spheroids) in the hydrogel (Fig. S1a). When iPSCs were initially seeded at 1.0 × 10^6^ cells/ml, 20-fold expansion could be achieved on day 5, generating ~2.0 × 10^7^ cells/ml. iPSCs could be cultured in the hydrogel scaffold for long term (above 10 passages) (Fig. S1c). The cell growth rate and doubling time (i.e. ~27 hours) were equal to these of cells cultured in Matrigel-coated 2D plates (Fig. S1c). Similar results were found for H9 cells (Fig. S1b,c). These results indicate the 3D PNIPAAm-PEG hydrogel is appropriate for hPSC culture and does not significantly change the cell behavior.

We then studied whether the hPSCs as spheroids could be differentiated into NSCs in the PNIPAAm-PEG hydrogel. Literature research has shown hPSCs cultured in 2D plates could be efficiently induced into neural stem cells (NSCs) by inhibiting the SMAD signaling under defined condition^49^. Our preliminary research confirmed these results. However, cells in the spheroids in the hydrogel face environments that are very different from these of the monolayer cells in the 2D cultures. We asked whether the 3D environments affected cell differentiation and the same differentiation protocol could be used to induce hPSCs (iPSCs and H9s) into NSCs in both 2D and 3D. To test this, we differentiated iPSCs into NSCs in parallel as monolayers on 2D surfaces and as spheroids in the 3D hydrogels. iPSC monolayers and spheroids with diameter around 100 μm were used for the 2D and 3D differentiation, respectively (Fig. 1a,b). To differentiate iPSCs into NSCs, bFGFs and TGFβs were removed from E8 medium and two small molecules LDN193189 (100 nM) and SB431542 (10 μM), which inhibit the SMAD signaling, were supplied to the medium. Very few dead cells were found in both 2D and 3D cultures during the differentiation as shown by the Live/Dead cell staining (Fig. 1a,b). On day 7, ~3.0 × 10^5^ cells/cm^2^ and 5.0 × 10^7^ cells/ml were yielded for 2D and 3D culture, respectively. Immunostaining demonstrated that >95% of the starting iPSCs expressed the pluripotency marker, OCT4 and NANOG. On day 7, less than 2% of the cells were positive for OCT4 and NANOG (Fig. 1c). 95% of the day 7 cells expressed high level of NSC markers, NESTIN and PAX6. There were no significant difference for the expressions of protein markers between the 2D and 3D differentiation (Fig. 1d,e).

We further compared the differentiation in 2D and 3D using qPCR (Fig. S2). qPCR data showed the pluripotency marker *OCT4* gradually decreased, while *NANOG* dropped sharply on day 1. NSC markers, such as *PAX6, NESTIN, FOXG1, SOX1* and *SOX2*, increased gradually. While there was no difference for the *PAX6, NESTIN* expressions between 2D and 3D differentiation, the 3D environment speeded up and enhanced the expressions of *FOXG1, SOX1*, and delayed the expression of *SOX2*. Similar results were found for the H9s (Figs S3 and S4). This comparative study indicates the small molecule-based differentiation protocol works efficiently in both 2D and 3D and the 3D environment does affect the expressions of some genes.

We then studied whether the hPSC-derived NSCs could be cultured *in vitro* to expand their numbers. We cultured the NSCs derived from iPSCs in both the 3D PNIPAAm-PEG hydrogels and 2D plates (Fig. 2 and S5). When NSCs made in the 3D hydrogels were dissociated and further cultured in 3D PNIPAAm-PEG hydrogels, they grew into spheroids and could be passaged every 5 days. However, at passage 4, significant cell death was seen as shown by the live/dead staining (Fig. 2a) and the culture could not be continued. When NSCs made in the 3D hydrogels were cultured on Matrigel-coated plates, cells were could be propagated at least 10 passages without significant cell death (Fig. 2b). Cells expressed the NSC markers, PAX6 and NESTIN, during the long term culture (Fig. 2c). However, qPCR data showed the transcript levels of *PAX6, NESTIN, FOXG1, SOX1* and *SOX2* decreased during the culture (Fig. 2d). NSCs derived from the iPSCs in 2D cultures (Fig. S5), and NSCs differentiated from H9s in both 2D cultures and 3D PNIPAAm-PEG hydrogels showed very similar results (Figs S6 and S7).

We studied whether iPSCs could be differentiated into regional NSCs in the 3D PNIPAAm-PEG hydrogel. Using dual SMAD inhibition without any patterning factors, the produced NSCs were default to be the precursor of cortical neurons^49^. When the day 11 NSCs released from the 3D hydrogels were cultured on Matrigel-coated plates for additional 19 days in neural differentiation medium, 95% of the cells became TUJ-1+ neurons. Among them, ~65% were TBR-1+ cortical neurons (Fig. 3a–c). qPCR showed the significant higher expressions of *TUJ-1* and *TBR-1* on day 30 than day 0 (Fig. 3d). If 0.7 μM CHIR99021 and 2 μM purmorphamine plus 200 ng/ml sonic hedgehog (shh) proteins were added to neural induction medium to activate the canonical Wnt and shh signaling, respectively, the NSCs could be patterned to the ventral midbrain fate as shown by the expression of ventral and midbrain markers: LMX1A and FOXA2 on day 11 (Fig. 3f)^15^. After maturing in the neural differentiation medium in 2D for additional 19 days, 92% of the cells were TUJ-1+ neurons. Among them, ~63% were TH+ midbrain neurons (Fig. 3g–i). Majority of these cells expressed the ventral and midbrain markers: LMX1A and FOXA2 (Fig. 3g). qPCR showed the significant higher expressions of *TUJ-1* and *TH* on day 30 than day 0 (Fig. 3h). These results indicate regional NSCs can be made from iPSCs in the 3D PNIPAAm-PEG hydrogels with pattern factors supplied to the medium.

**Figure 4:**
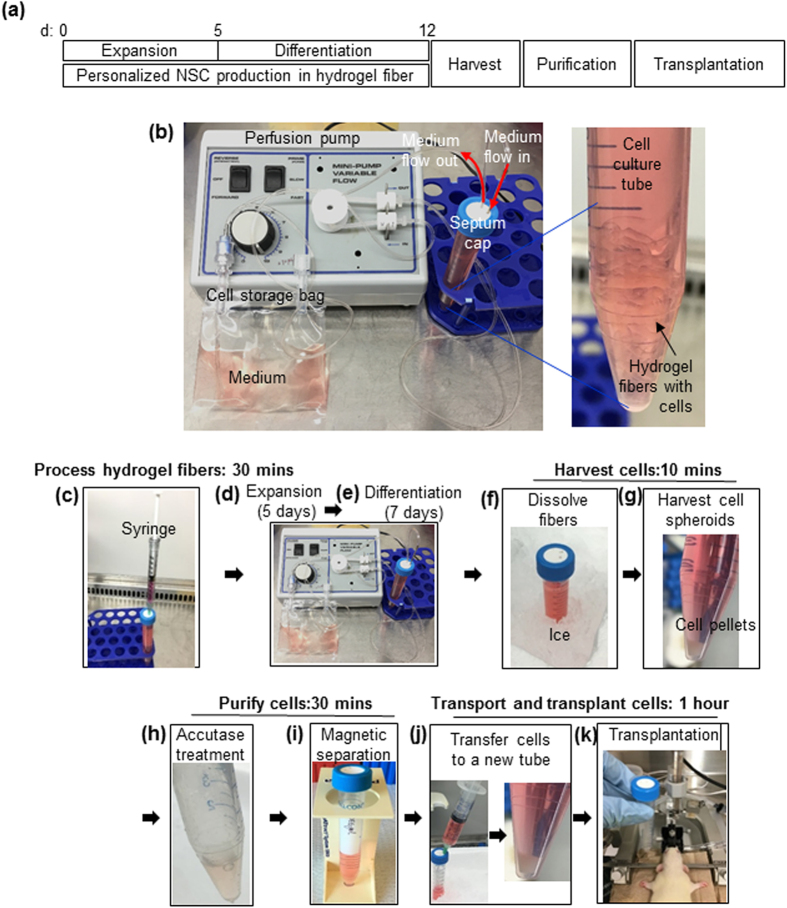
An integrated miniature bioprocessing for iPSC expansion and differentiation into NSCs. (**a**) Illustration of the bioprocessing. (**b**) The miniature cell culture device consists of a pump for medium perfusion, an oxygen-permeable plastic bag for stocking medium and a closed 15 ml conical tube. Fibrous hydrogel fibers with cells were suspended in the tube. (**c**) On day 0, single iPSCs were mixed with 10% PNIPAAm-PEG solution at 4 °C. With a syringe and needle, the mixture was injected into room temperature E8 medium contained in a closed and sterile 15 ml conical tube with a septum cap. Fibrous hydrogels (with diameter <2.0 mm) were instantly formed. (**d,e**) The cells were cultured in E8 medium for 5 days, followed by additional 7 days of neural induction medium in the conical tube. Medium was continuously perfused. (**f**) On day 12, hydrogel scaffolds were liquefied by placing the cell culture tube on ice for 5 minutes. (**g**) Cell spheroids were pelleted by spinning the tube at 100 g for 3 minutes. Medium was removed. (**h**) Cell spheroids were incubated in Accutase at 37 °C for 10 minutes. Removing reagents from the tube and adding reagents to the tube were done with a sterile syringe through the septum cap. (**i**) Magnetic beads coated with anti-SSEA4 antibodies were added into the tube to pull down the undifferentiated SSEA4+ iPSCs with a magnetic cell separator. (**j**) Purified cells in the supernatant were transferred into a new, close tube and transported to the surgical room. (**k**) NSCs were transplanted to the rat brain with a stereotactic injector.

We then took advantage of the high cell yield in the PNIPAAm-PEG hydrogels to build a prototype miniature bioprocessing for making NSCs from iPSCs for personalized cell therapies (Fig. 4). On day 0, single iPSCs were mixed with 10% PNIPAAm-PEG solution at 4 °C. With a syringe and needle, the mixture was injected into room temperature E8 medium contained in a closed and sterile 15 ml conical tube with a septum cap (Fig. 4c). Fibrous hydrogels (with diameter <2.0 mm) were instantly formed with single iPSCs uniformly distributed in the hydrogels. The cells were cultured in a cell culture incubator at 37 °C and 5% CO_2_. Medium stocked in a gas-permeable bag was continuously perfused into the cell culture tube (Fig. 4b). E8 medium was supplied for 5 days (Fig. 4d), followed by additional 7 days of neural induction medium (Fig. 4e). On day 7, hydrogel scaffolds were liquefied by placing the cell culture tube on ice for 5 minutes (Fig. 4f). Cell spheroids were pelleted by spinning the tube at 100 g for 3 minutes (Fig. 4g). Medium was removed. Cell spheroids were incubated in Accutase at 37 °C for 10 minutes (Fig. 4h). Removing reagents from the tube and adding reagents to the tube were done with a sterile syringe through the septum cap. Magnetic beads coated with anti-SSEA4 antibodies were added into the tube to pull down the undifferentiated SSEA4+ iPSCs with a magnetic cell separator (Fig. 4i). Purified cells in the supernatant were transferred into a new, close tube (Fig. 4j) and transported to the surgical room. NSCs were transplanted to the brains of Sprague dawley rats with a stereotactic injector (Fig. 4k).

**Figure 5:**
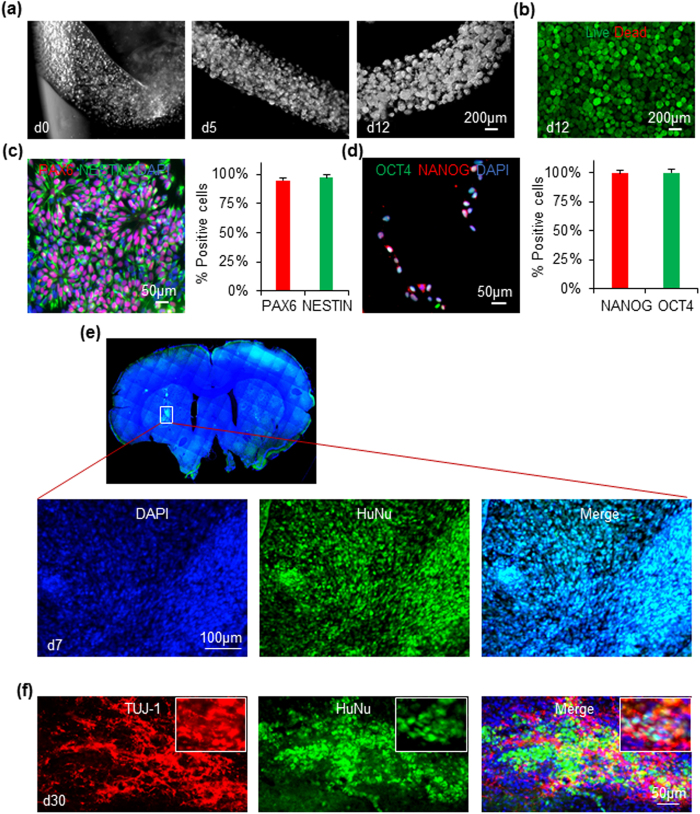
Cells in the miniature bioprocessing. (**a**) Phase images of the hydrogel fibers and cells on day 0, 5 and 12 of the bioprocessing. (**b**) Live/dead staining of cells on day 12. (**c**) ~97% of the purified cell products expressed NSC markers, PAX6 and NESTIN. (**d**) Cells pull down by the magnetic anti-SSEA4 beads were positive for OCT4 and NANOG. (**e**) HuNu+ (human nuclear antigen) NSCs survived well in the rat brain 7 days post-transplantation. (**f**) Substantial amounts of transplanted NSCs became HuNu+ and TUJ-1+ neurons in the rat brain 30 days post-transplantation.

Single iPSCs in hydrogel fibers grew into iPSC spheroids on day 5, and then became NSC spheroids on day 12 (Fig. 5a). With initial seeding density at 1 × 10^6^ cells/ml, 25-fold expansion and 2.5 × 10^7^ cells/ml hydrogel were achieved on day 12. Total of 1.0 × 10^8^ cells were produced in 4 ml of hydrogel in a 15 ml conical tube. Cell viability was >95% on day 12. 2% of the day 12 cells were SSEA4+. Live/dead cell staining showed no or undetectable dead cells (Fig. 5b). After magnetic separation, the produced cells expressed PAX6 and NESTIN (Fig. 5c) and OCT4+/NANOG+ cells were not detectable. Cells pull down by the magnetic beads expressed both OCT4 and NANOG (Fig. 5d). 7 days post-transplantation, substantial numbers of human cells, as shown by the expression of human nuclear antigen (HuNu+) were found in the rat brain (Fig. 5e). 30 days post-transplantation, substantial numbers of human neurons (e.g. HuNu+ and TUJ-1+) were found in the rat brain (Fig. 5f).

## Discussion

Transgene free iPSCs can be readily made from adults cells with the most recent integration free reprogramming vectors^6^. iPSCs can be expanded *in vitro* to generate large numbers of cells. They can also be differentiated presumably all the human cell types. Additionally, there are no ethic debates with using iPSCs derived products. Thus, iPSCs and their derivatives have enormous potential to treat human diseases, either by transplanting them to replace the lost cells or to alter the disease progression through stand by mechanisms, or using them to engineer tissues or organs to replace the dysfunctional ones^1^,^2^,^3^,^4^,^5^.

There are two approaches to use iPSC-derived products. iPSCs and their derivatives can be manufactured in large scale and used as off-shelf products^50^. The advantages of this approach include: (a) off-shelf products can be available when the patients need them, and (b) the large-scale production reduces the biomanufacturing cost. However, allogenic cells usually induce severe immune response. A strategy to minimize the immune response is to generate libraries of iPSCs to provide human leukocyte antigen (HLA)-matched cells to majority of the patients^51^,^52^,^53^. However, the cost and effort to establish these libraries are not trivial^51^,^52^,^53^. In addition, HLA-matched cells may still induce immune response^1^,^2^,^54^. The second approach is to make patient-specific iPSC products^1^,^2^,^54^. Research has shown patient-specific iPSC products induce no or minimal immune response. However, the cost for biomanufacturing personalized iPSCs and their derivatives with current technologies are extremely high and not affordable for majority of patients^1^,^2^,^3^,^4^,^5^.

Automating the bioprocessing in individualized, closed, computer controlled miniature cell culture devices can significantly reduce the biomanufacturing cost^42^. However, it is very challenging to develop such type of cell culture device with current 2D cell culture (e.g. cell culture dishes) and 3D suspension culture (e.g. spinner flasks) technologies^44^. The low yield and frequent cell passaging make the 2D culture systems not a good choice as personalized cellular biomanufacturing system. In 3D suspension cultures, cells frequently aggregate into large cell agglomerates^43^,^44^,^46^. Due to the diffusion limits, the supply of growth factors, nutrients and oxygen to and the depletion of the metabolic waste from the cells located at the center of cell agglomerates became insufficient, leading to slow cell proliferation, apoptosis and uncontrolled differentiation. Stirring or shaking is widely used to reduce the cellular agglomeration and enhance mass transport in suspension cultures^44^. However, it also generated hydrodynamic stress that negatively affects the cell growth and quality^55^,^56^. High cell density in the culture also promotes spheroid agglomeration^46^. Considering all these factors, cells are generally seeded at low density (e.g. ~5.0 × 10^5^ cells/ml) and stirred at 70 to 120 rpm in suspension cultures. Even under these optimized conditions, cells grow slowly and cell death is common. For instance, iPSCs typically expand 4 times per 4 days to yield around 2.0 × 10^6^ cells/ml in spinner flasks^46^. It is good to note that cells occupy <0.4% of the medium volume with this yield. It would require a 50 ml to 500 ml of 3D suspension cultures to produce 1 × 10^8^ to 1 × 10^9^ cells^44^. This low volumetric yield makes the 3D suspension culture systems not attractive for developing miniature cell culture devices.

A previous study showed hPSCs could be cultured in thermoreversible PNIPAAm-PEG hydrogels at much higher efficiency^46^. The hydrogel scaffolds provided 3D spaces for cell growth and also acted as physical barriers to prevent cell agglomeration and isolate the hydrodynamic stresses. The high cell growth rate, yield and quality makes this hydrogel very attractive for developing personalized cell culture device. In this paper, we demonstrated that personalized NSCs could be made from iPSCs within a miniature cell culture device with the assist of the PNIPAAm-PEG hydrogels (Figs 4 and 5). We showed that: (a) the whole bioprocess including iPSC expansion, iPSC differentiation into NSCs, the product purification, concentration and transport to the surgery room, could be completed with two closed conical tubes. In this study, we depleted the undifferentiated iPSCs with anti-SSEA4 magnetic beads and the purified NSCs were transferred to a second tube (Figs 4 and 5). An alternative approach is to positively isolate the NSCs with magnetic beads coated with antibodies recognizing NSCs. With this approach, the whole bioprocess can be done with one tube; (b) ~1.0 × 10^8^ high purity NSCs could be made with 4 ml hydrogel scaffolds within a 15 ml conical cell culture tube. It is calculated that 1 × 10^9^ cells, which are needed to treat many human diseases, can be made with 40 ml of fibrous hydrogel scaffolds in a 50 ml conical tube. This miniaturized device allows high throughput biomanufacturing of personalized cells for large numbers of patients at the point-of-care; (c) majority of the bioprocessing could be done automatically. In the future, processing fibers, harvesting, purifying and concentrating cells will also be automated. Recent research demonstrated reprogramming human adult cells into iPSCs could be efficiently done in 3D hydrogel scaffolds^57^. And our preliminary research also showed iPSCs could be made from fibroblasts in the 3D PNIPAAm-PEG hydrogels. In the future, we will also integrate the reprogramming into the miniaturized bioprocessing within the conical tube.

iPSCs were differentiated into NSCs to demonstrate the personalized cellular biomanufacturing system in this paper. iPSC-derived NSCs are being widely studied for treating various human neuronal diseases^58^,^59^. These NSCs could survive, mature and outgrowth long nerve fibers in rodent model of spinal cord injuries^60^. iPSC-derived NSCs patterned to the forebrain cortical fate could survive and significantly improve the function of stroked brains in rodents^33^. iPSC-derived NSCs could migrate toward brain tumors and thus are being studied as gene or drug delivery vehicles for treating brain cancers^61^. NSCs isolated from fetal brains have been in clinical trials for treating a few rare neuronal degenerative diseases^62^. iPSC-derived NSCs have potential to replace these fetal NSCs to avoid the ethical issues and host immune response.

The protocols for differentiating hPSCs into NSCs in the literature are developed in 2D culture systems and may be not applicable for differentiating hPSCs as spheroids in the hydrogel scaffolds. The cell spheroids may have increased amounts of extracellular proteins, cell-cell, cell-matrix interactions^63^. The concentrations of many protein factors in the 3D cell mass may be different from these in the 2D cell monolayers. In addition, gradients may exist in the 3D cell mass and hydrogel. The transport of medium components and metabolic waste in the cell spheroids and hydrogel scaffold are also different from these in the 2D cultures. We systematically compared the iPSC differentiation into NSCs as spheroids in the 3D hydrogel and as monolayers in 2D. In general, the small molecules-based differentiation protocol used in this research worked efficiently in both 3D and 2D cultures (Fig. 1 and S3). However, the transcripts of SOX1 and FOXG1 appeared earlier and stronger in 3D, while the transcript of SOX2 was delayed in 3D (Figs S2 and S4). Literature research has shown that, during the neural differentiation of hPSCs as embryoid body, hPSCs first became primitive neuroepithelia NSCs expressing PAX6 and SOX2, but no SOX1, then became definitive neuroepithlia NSCs expressing PAX6, SOX2 and SOX1^64^,^65^. Our results showed NSCs generated in 2D cultures and in 3D hydrogel might be mainly primitive and definitive neuroepithelia NSCs, respectively, and the 3D environment promoted forebrain fate NSCs, as shown by the high level of forebrain marker FOXG1 (Fig. S2)^66^. Our research showed that NSCs derived from hPSCs in both 3D and 2D could be cultured for long term in 2D, but not in 3D hydrogels (Fig. 2 and S5). In addition, the transcript level of some genes that are important for NSCs were altered during the long term culture (Fig. 2 and S6).

In summary, we showed that, with the assist of appropriate hydrogel scaffolds, personalized iPSC expansion and differentiation, as well as the subsequent product purification could be integrated into miniature bioprocessing that can be automated. We believe this type of bioprocessing can help the advancement of iPSC-based personalized cell therapies. To our best knowledge, this is the first report on hydrogel-based miniature cell culture device/bioprocessing for personalized iPSC expansion and differentiation.

## Additional Information

**How to cite this article**: Lin, H. *et al*. An Integrated Miniature Bioprocessing for Personalized Human Induced Pluripotent Stem Cell Expansion and Differentiation into Neural Stem Cells. *Sci. Rep.* **7**, 40191; doi: 10.1038/srep40191 (2017).

**Publisher's note:** Springer Nature remains neutral with regard to jurisdictional claims in published maps and institutional affiliations.

## Supplementary Material

Supplementary Figures
