# Supplementary material for: An Integrated Miniature Bioprocessing for Personalized Human Induced Pluripotent Stem Cell Expansion and Differentiation into Neural Stem Cells
Source: Sci Rep. 2017 Jan 6;7:40191. doi: 10.1038/srep40191 (PMC5216399; doi:10.1038/srep40191)
Supplement: Supplementary Figures [file srep40191-s1.pdf]

Supporting information

An Integrated Miniature Bioprocessing for Personalized Human Induced Pluripotent Stem Cell Expansion and Differentiation into Neural Stem Cells

Haishuang Lin[1], Qiang Li[1] and Yuguo Lei[1,2,3,*]

1: Department of Chemical and Biomolecular Engineering, University of Nebraska, Lincoln, Nebraska, USA

2: Mary and Dick Holland Regenerative Medicine Program, University of Nebraska Medical Center, Omaha, Nebraska, USA

3: Fred & Pamela Buffett Cancer Center, University of Nebraska Medical Center, Omaha, Nebraska, USA

* Corresponding Author

Yuguo Lei

820 N 16th St

Lincoln, NE 68588

Email : ylei14@unl.edu

Phone: 402-472-5313
Fax: 402-472-6989

a

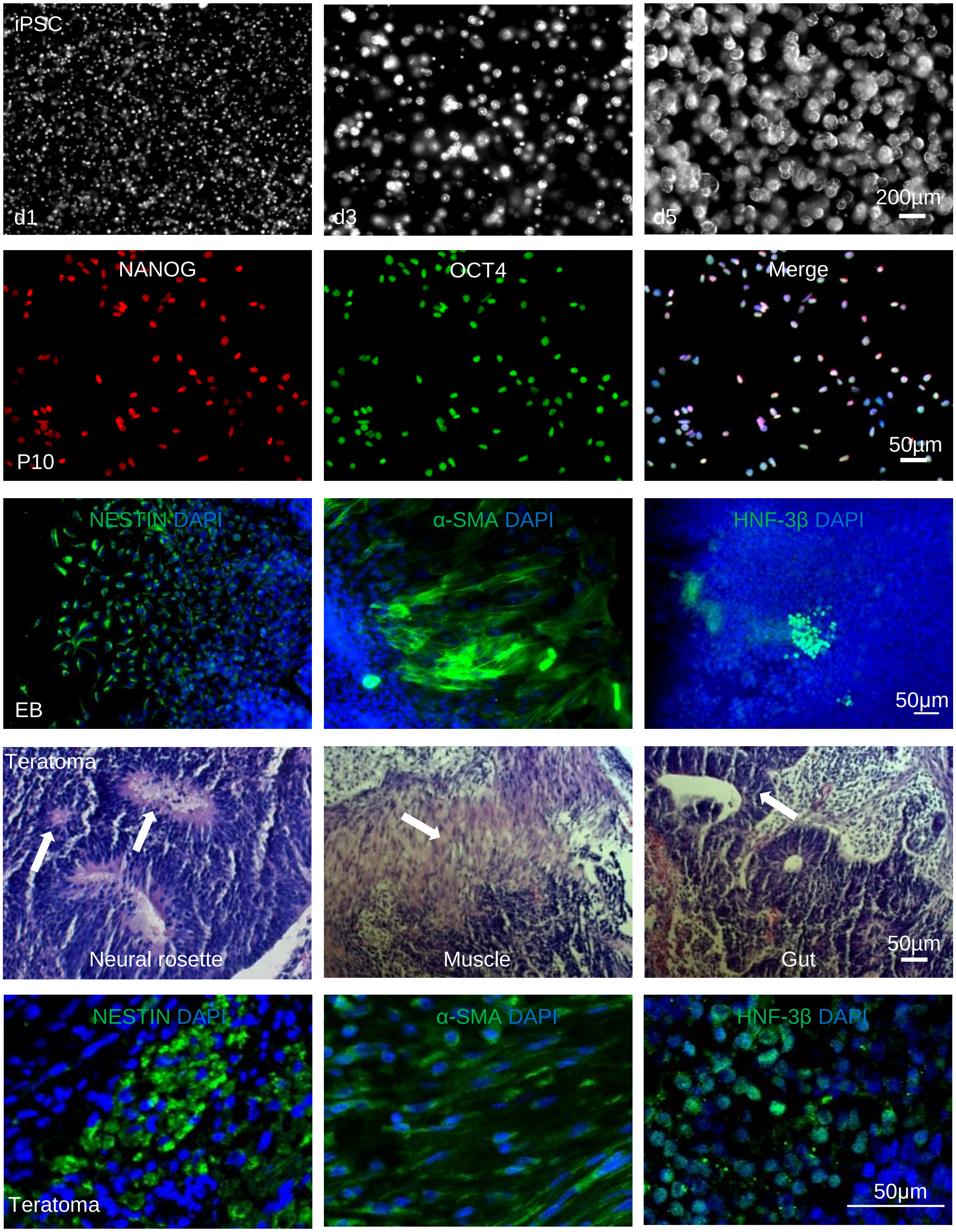
iPSC
d1
d3
d5
200µm
NANOG
OCT4
Merge
P10
50µm
NESTIN DAPI
α-SMA DAPI
HNF-3β DAPI
EB
50µm
Teratoma
Neural rosette
Muscle
Gut
50µm
NESTIN DAPI
α-SMA DAPI
HNF-3β DAPI
Teratoma
50µm

b

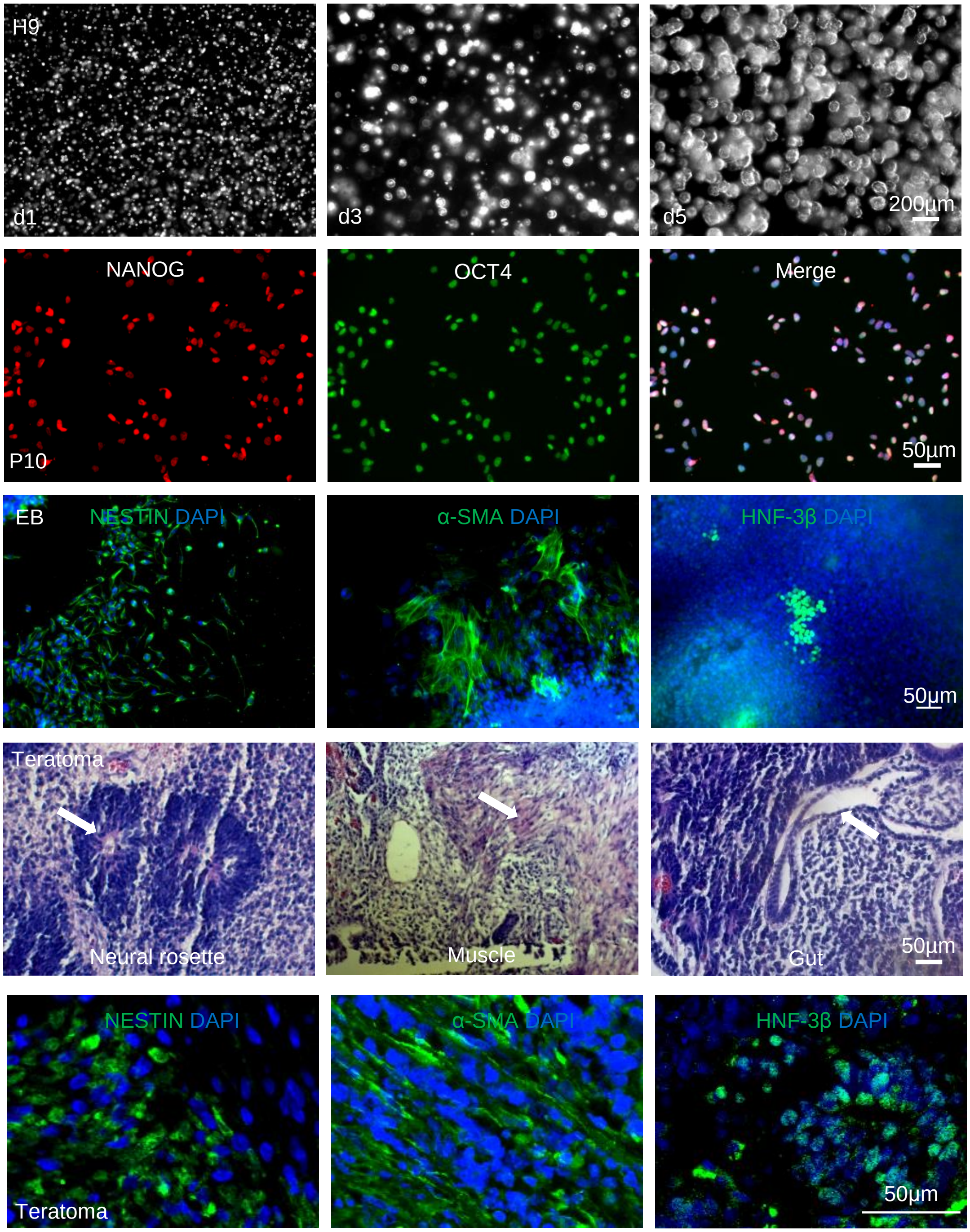

H9
d1
d3
d5
200µm
NANOG
OCT4
Merge
P10
50µm
EB
NESTIN DAPI
α-SMA DAPI
HNF-3β DAPI
50µm
Teratoma
Neural rosette
Muscle
Gut
50µm
NESTIN DAPI
α-SMA DAPI
HNF-3β DAPI
Teratoma
50µm

c

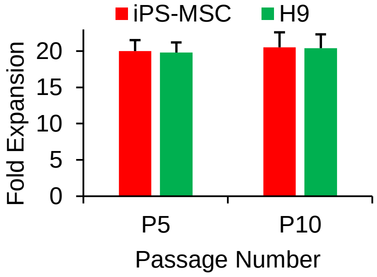

iPS-MSC
H9
Fold Expansion
20
15
10
5
0
P5
P10
Passage Number

Figure S1. Long term culture of iPSCs (a) and H9s (b) in the 3D PNIPAAm-PEG hydrogels. Phase images showed single cells grew into uniform spheroids in 5 days. After 10 passages, majority of the cells expressed the pluripotency markers, OCT4 and NANOG. The long-term cultured hPSCs could be differentiated into all the three germ layer cells, including NESTIN+ ectodermal, α-SMA+ mesodermal and HNF-3β+ endodermal cells in the EB assay. In NOD-SCID mice, these long-term cultured hPSCs formed teratomas containing all the three germ layers tissues (arrows) and all the three germ layer cells, including NESTIN+ ectodermal, α-SMA+ mesodermal and HNF-3β+ endodermal cells. In general, cells expanded 20-fold per 5 days (c), yielding 2.0×10^7 cells/ml.

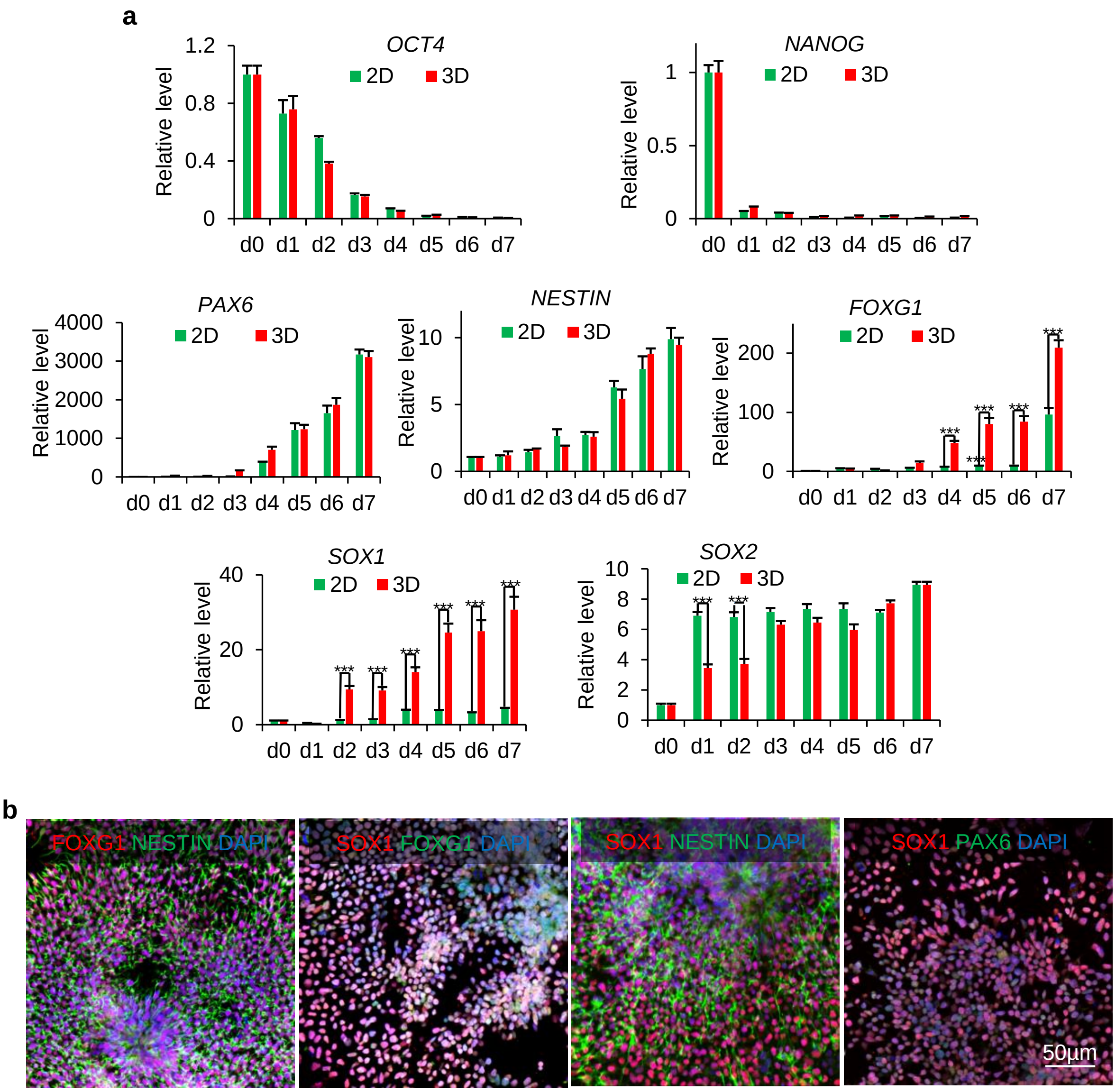

Figure S2. Differentiation of human iPSCs into NSCs in 2D and 3D hydrogels. (a) qPCR showing the expressions of pluripotency and NSC genes along the 7 days differentiation. *** indicates statistical significance at a level of $p<0.001$. (b) Immunostaining of NSC markers including PAX6, NESTIN, SOX1 and FOXG1 on NSCs generated in the 3D thermoreversible hydrogel.

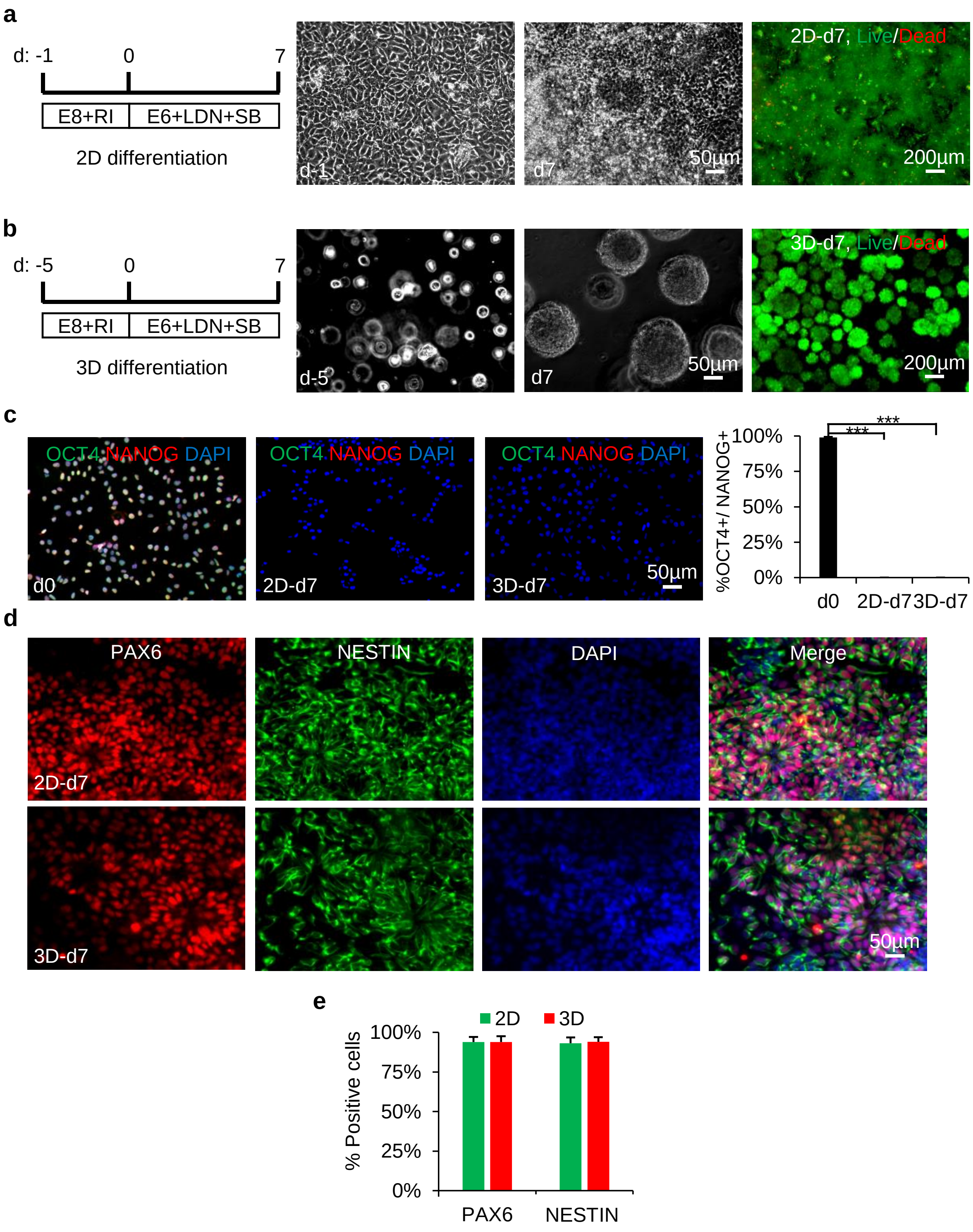
a
d: -1
0
7
E8+RI
E6+LDN+SB
2D differentiation
d-1
d7
50µm
2D-d7, Live/Dead
200µm
b
d: -5
0
7
E8+RI
E6+LDN+SB
3D differentiation
d-5
d7
50µm
3D-d7, Live/Dead
200µm
c
OCT4 NANOG DAPI
d0
OCT4 NANOG DAPI
2D-d7
OCT4 NANOG DAPI
3D-d7
50µm
%OCT4+/ NANOG+
100%
75%
50%
25%
0%
***
***
d0
2D-d7
3D-d7
d
PAX6
NESTIN
DAPI
Merge
2D-d7
3D-d7
50µm
e
2D
3D
% Positive cells
100%
75%
50%
25%
0%
PAX6
NESTIN

Figure S3. Differentiation of H9s into NSCs in 2D cultures and 3D PNIPAAm-PEG hydrogels. For differentiation in 2D (a), single H9s were plated on Matrigel-coated plate overnight to reach 90% confluency. For differentiation in 3D (b), single H9s were cultured in the PNIPAAm-PEG hydrogel for 5 days to generate uniform spheroids. Cells were differentiated in neutral induction medium for 7 days. Phase images for cells on day 0 (d0) and 7 (d7), as well as live/dead cell staining for cells on day 7 are shown. (c) 98% of cells expressed the pluripotency markers, OCT4 and NANOG before the differentiation. Less than 2% of cells were OCT4+/NANOG+ after 7 days differentiation in 2D or 3D. Cells were dissociated into single cells and plated for 6 hours before the fixation and staining. (d, e) On day 7, ~95% of cells were positive for the NSC markers, PAX6 and NESTIN. The 3D spheroids were plated on Matrigel-coated plate overnight to form monolayers before the fixation and staining. The triple asterisk (***) indicates statistical significance at a level of $p<0.001$.

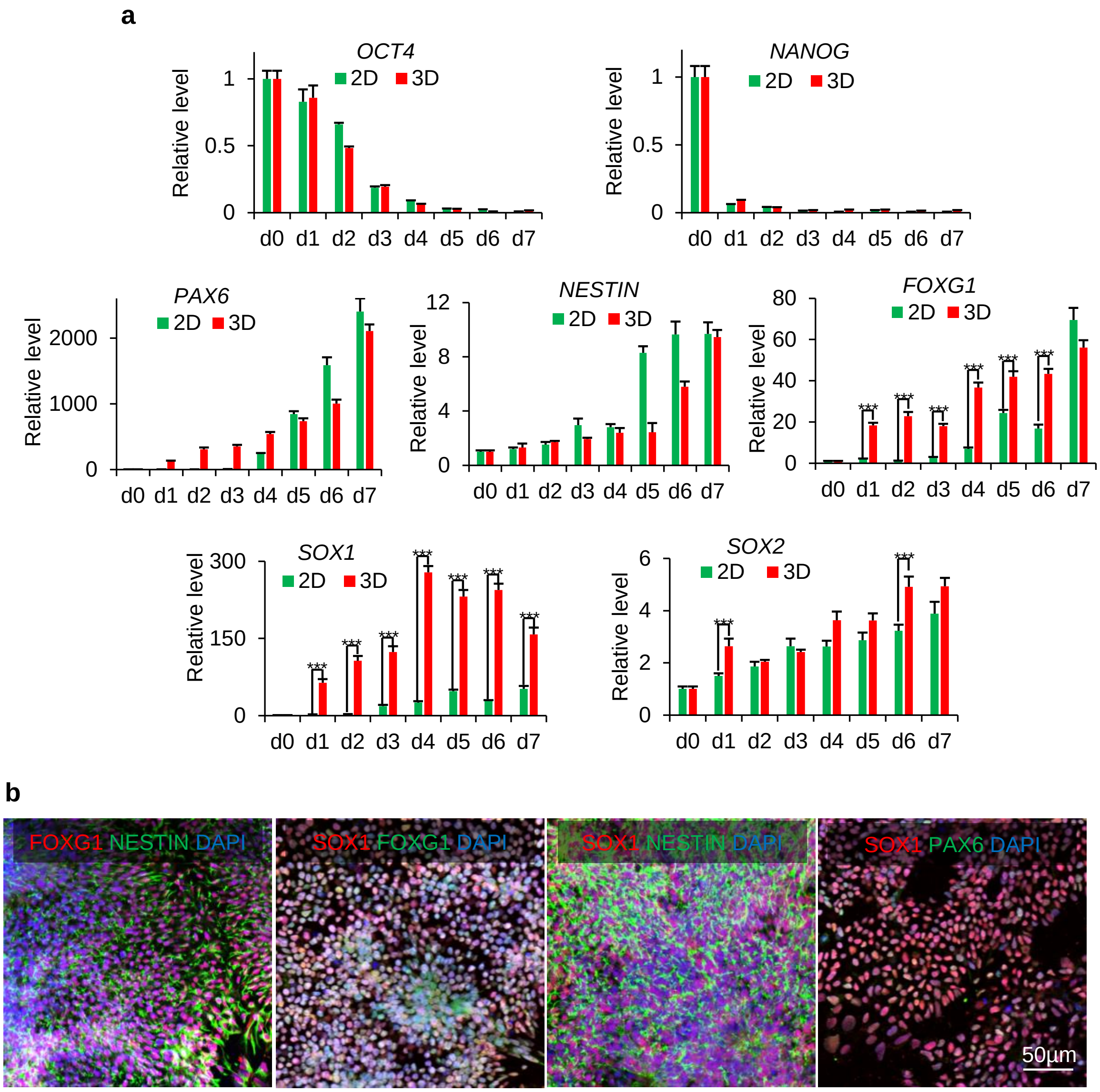

Figure S4. Differentiation of human H9s into NSCs in 2D and 3D hydrogels. (a) qPCR showing the expressions of pluripotency and NSC genes along the 7 days differentiation. *** indicates statistical significance at a level of p<0.001. (b) Immunostaining of NSC markers including PAX6, NESTIN, SOX1 and FOXG1 on NSCs generated in the 3D thermoreversible hydrogel.

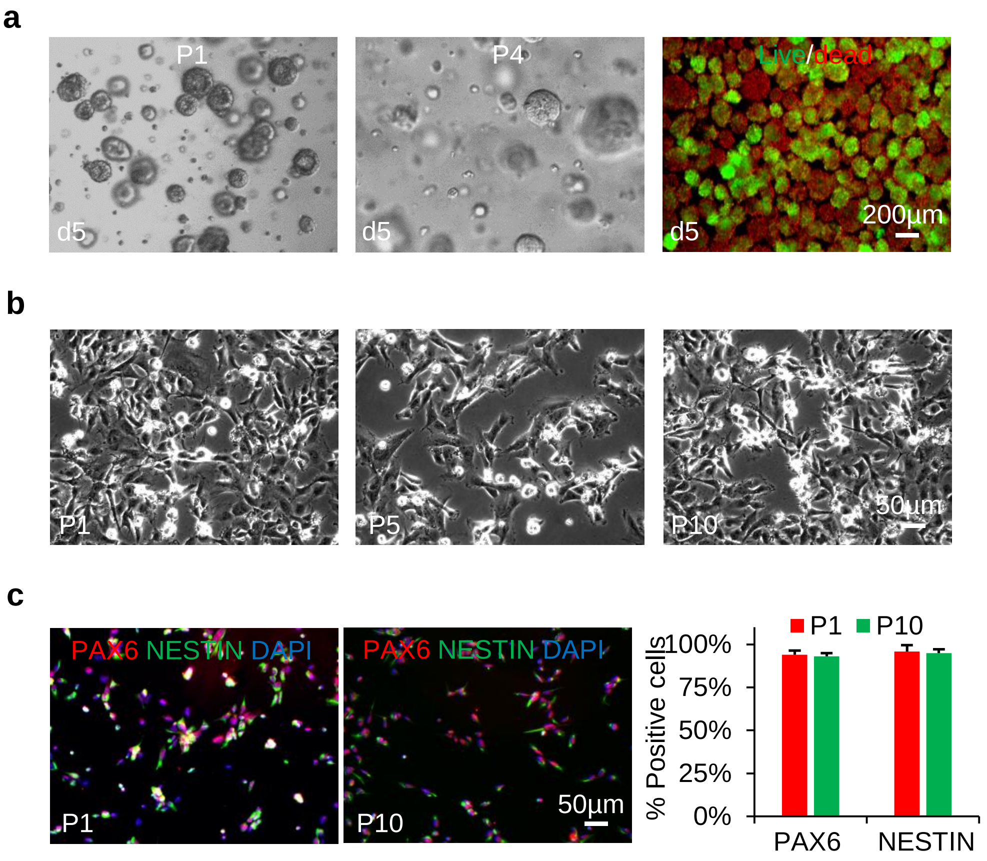

Figure S5. Culturing iPSC-derived NSCs in 3D and 2D. NSCs derived from iPSCs in 2D cultures were dissociated and cultured in the 3D PNIPAAm-PEG hydrogel (a) or on Matrigel-coated 6 well plate (b-c). Cells were passaged every 5 days or 3 days for 3D and 2D cultures, respectively. (a) Phase and live/dead staining images of day 5 spheroids at passage 1 (P1) and 4 (P4) in the 3D PNIPAAm-PEG hydrogels. (b) Phase images of NSCs on day 3 of passage 1, 5 and 10 (P1, P5, P10) in 2D cultures. (c) 90-95% of the NSCs at P1 and P10 in 2D cultures were positive for the NSC markers, PAX6 and NESTIN.

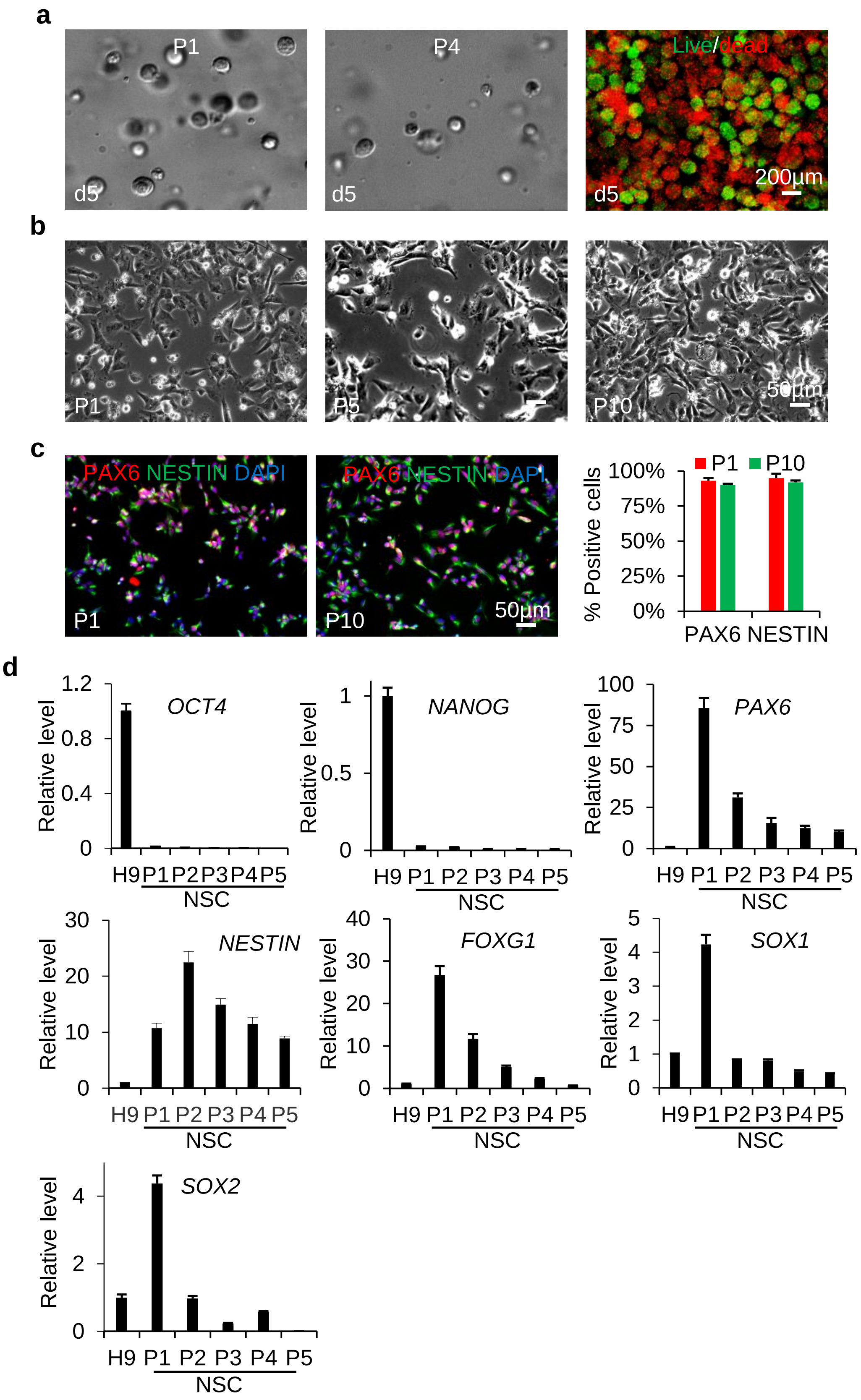
a
P1
d5
P4
d5
Live/dead
200μm
d5
b
P1
P5
P10
50μm
c
PAX6 NESTIN DAPI
P1
PAX6 NESTIN DAPI
P10
50μm
% Positive cells
100%
75%
50%
25%
0%
P1
P10
PAX6 NESTIN
d
Relative level
1.2
0.8
0.4
0
OCT4
H9 P1 P2 P3 P4 P5
NSC
Relative level
1
0.5
0
NANOG
H9 P1 P2 P3 P4 P5
NSC
Relative level
100
75
50
25
0
PAX6
H9 P1 P2 P3 P4 P5
NSC
Relative level
30
20
10
0
NESTIN
H9 P1 P2 P3 P4 P5
NSC
Relative level
40
30
20
10
0
FOXG1
H9 P1 P2 P3 P4 P5
NSC
Relative level
5
4
3
2
1
0
SOX1
H9 P1 P2 P3 P4 P5
NSC
Relative level
4
2
0
SOX2
H9 P1 P2 P3 P4 P5
NSC

Figure S6. Culturing H9-derived NSCs in 3D PNIPAAm-PEG hydrogels and 2D cultures. NSCs derived from H9s in the 3D PNIPAAm-PEG hydrogel were dissociated and cultured in the 3D PNIPAAm-PEG hydrogel (a) or on Matrigel-coated 6 well plate (a-d). Cells were passaged every 5 days or 3 days for 3D and 2D cultures, respectively. (a) Phase and live/dead staining images of day 5 spheroids at passage 1 (P1) and 4 (P4) in the 3D PNIPAAm-PEG hydrogels. (b) Phase images of NSCs on day 3 of passage 1, 5 and 10 (P1, P5, P10) in 2D cultures. (c) 90-95% of the NSCs at P1 and P10 in 2D cultures were positive for the NSC markers, PAX6 and NESTIN. (d) qPCR data on the expressions of pluripotency and NSC markers during P1 to P5 in 2D cultures.

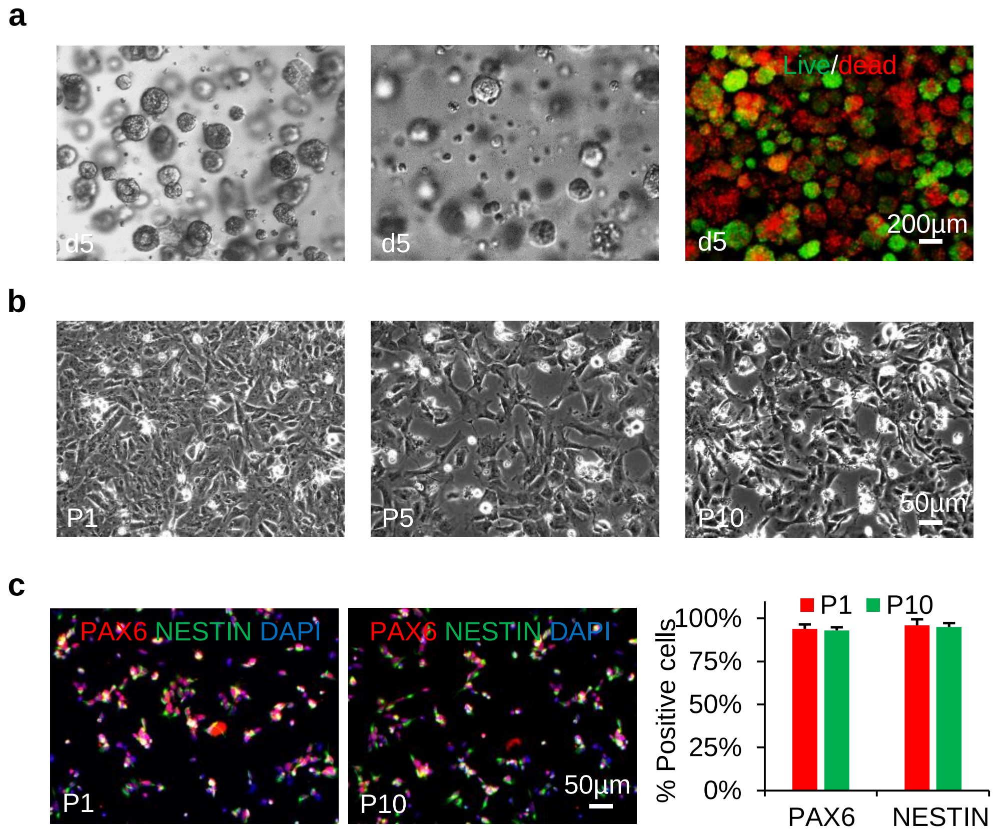

Figure S7. Culturing H9-derived NSCs in 3D and 2D. NSCs derived from H9s in 2D cultures were dissociated and cultured in the 3D PNIPAAm-PEG hydrogel (a) or on Matrigel-coated 6 well plate (b-c). Cells were passaged every 5 days or 3 days for 3D and 2D cultures, respectively. (a) Phase and live/dead staining images of day 5 spheroids at passage 1 (P1) and 4 (P4) in the 3D PNIPAAm-PEG hydrogels. (b) Phase images of NSCs on day 3 of passage 1, 5 and 10 (P1, P5, P10) in 2D cultures. (c) 90-95% of the NSCs at P1 and P10 in 2D cultures were positive for the NSC markers, PAX6 and NESTIN.

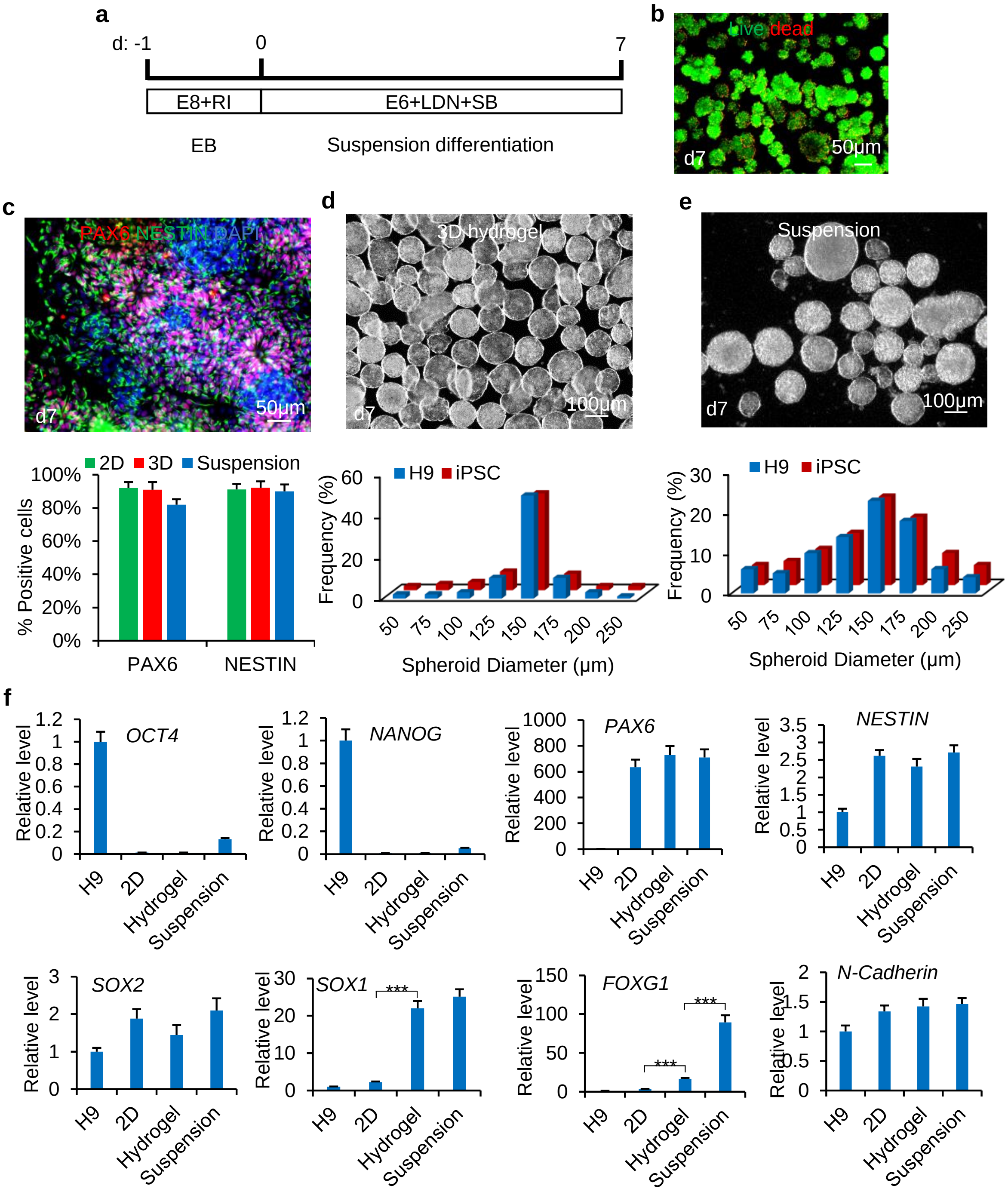
a
d: -1
0
7
E8+RI
E6+LDN+SB
EB
Suspension differentiation
b
Live dead
d7
50μm
c
PAX6 NESTIN DAPI
d7
50μm
d
3D hydrogel
d7
100μm
e
Suspension
d7
100μm
2D 3D Suspension
% Positive cells
PAX6
NESTIN
H9 iPSC
Frequency (%)
Spheroid Diameter (μm)
f
Relative level
OCT4
NANOG
PAX6
NESTIN
SOX2
SOX1
FOXG1
N-Cadherin
H9
2D
Hydrogel
Suspension
***

Figure S8. Comparison of neural differentiation as 2D adherent cells, non-adherent embryoid bodies (EBs) in suspension and spheroids in 3D hydrogel. (a) Schematic illustration of neural differentiation as non-adherent embryoid bodies (EBs). Briefly, single hPSCs were suspended in the medium in low-attached 6-well plate on day -1 to form EBs. Neural differentiation was started on day 0. The protocols for neural differentiation as 2D adherent cells and spheroids in 3D hydrogel were illustrated in Figure 1 and described in details in the Methods. The medium formulations for all the three differentiations were identical. (b) Live/dead staining revealed large numbers of dead cells in the EBs on day 7. (c) Immunostaining showed more cells in the 2D adherent cells and spheroids in 3D hydrogel than in the EBs expressed NSC marker PAX6. (d, e) While the day 7 spheroids in the 3D hydrogel had uniform size, the day 7 EBs had much broader size distribution. (f) RT-PCR analysis of the expression of hPSC- and NSC-associated genes in 2D adherent cells, non-adherent embryoid bodies (EBs) and spheroids in 3D hydrogel on day 7.
